# Andrographolide Alleviates Acute Brain Injury in a Rat Model of Traumatic Brain Injury: Possible Involvement of Inflammatory Signaling

**DOI:** 10.3389/fnins.2018.00657

**Published:** 2018-09-20

**Authors:** Li Tao, Li Zhang, Rong Gao, Feng Jiang, Jianbo Cao, Huixiang Liu

**Affiliations:** ^1^Department of Pharmacy and Translational Medicine Center, Zhangjiagang First People’s Hospital, Suzhou, China; ^2^Department of Neurosurgery, Zhangjiagang First People’s Hospital, Suzhou, China

**Keywords:** andrographolide, inflammation, apoptosis, traumatic brain injury, neuroprotection

## Abstract

Neuroinflammation plays an important role in secondary injury after traumatic brain injury (TBI). Andrographolide (Andro), a diterpenoid lactone isolated from *Andrographis paniculata*, has been demonstrated to exhibit anti-inflammatory activity in neurodegenerative disorders. This study therefore aimed to investigate the potential neuroprotective effects of Andro after TBI and explore the underlying mechanisms. In our study, we used a weight-dropped model to induce TBI in Sprague–Dawley rats, the neurological deficits were assessed using modified neurological severity scores, Fluoro-Jade B (FJB) and terminal deoxynucleotidyl transferase (TdT) dUTP Nick-End Labeling (TUNEL) staining were employed to examine neuronal degeneration and apoptosis after TBI, immunofluorescence was designed to investigate microglial activation. Quantitative Real-time PCR and ELISA were conducted to detect the expression levels of pro-inflammatory cytokines, Western blot was used to examine the expression level of proteins of relative signaling pathway. Our results showed that after Andro administration, the neurological deficit was attenuated, and the cerebral edema and apoptosis in brain tissues were also decreased following TBI. Both microglial activation and the expression of pro-inflammatory cytokines were significantly inhibited by Andro after TBI. Moreover, Andro inhibited NF-κB p65 subunit translocation and decreased the expression levels of phosphorylated extracellular signal regulated kinase (ERK) and p38 MAPK after TBI. Altogether, this study suggests that Andro could improve neurobehavioral function by inhibiting NF-κB and MAPK signaling pathway in TBI, which might provide a new approach for treating brain injury.

## Introduction

Traumatic brain injury (TBI) is a prevalent healthcare concern with more than 10 million people suffering annually worldwide. It has been a major cause of mortality and disability resulting from traffic accidents, falls, and external mechanical concussion etc, especially in children and young adults (Hyder et al., 2007; Gardner and Zafonte, 2016). TBI can lead to temporary or permanent motor deficit and cognition deficits which negatively affects the quality of life of patients. Despite the progress in diagnosis, neuroradiology, neurosurgical care and treatment in recent years, effective treatment strategies for improving functional outcome of TBI patients remain limited. Therefore, it is important to develop novel therapeutic interventions that could be helpful for TBI patients.

The pathophysiology of TBI can be divided with two parts: primary and secondary injury. Primary brain injury involve brain contusion, skull fractures, diffused axonal injury and intracranial hemorrhage, usually causes immediate neuronal death (Laplaca et al., 2007; Cheng et al., 2012). Secondary brain injury is a complicated cellular processes and biochemical cascades which occur in minutes and last for days following the traumatic events, resulting in exacerbated damage, progressive neurodegeneration and cell death (Kabadi and Faden, 2014). The mechanism of TBI-induced secondary injury includes glutamate excitotoxicity (Hyder et al., 2007), oxidative stress (Zhang et al., 2018), blood-brain barrier (BBB) disruption (Lutton et al., 2017), neuroinflammation and mitochondrial dysfunction (Lozano et al., 2015). It is well known that neuroinflammation occurs in both primary and secondary injury following TBI, as well as in other neurodegenerative diseases (Eikelenboom et al., 2010; Perry et al., 2010). As the resident immune cells in brain, microglia plays important roles in response to neuronal pathology. Activated microglia release a complex series of pro-inflammatory factors such as nitric oxide (NO), tumor necrosis factor (TNF), prostaglandin E2 (PGE2) and, interleukin (IL), leading to detrimental effects and neuronal dysfunction (Chiu et al., 2016). Therefore, inhibiting microglial activation might be a beneficial therapeutic target for treating TBI patients.

Andrographolide (Andro) is a natural diterpenoid from Chinese traditional herb *Andrographis paniculata*, which is widely used for treating fever, upper respiratory tract infections, laryngitis, diarrhea and rheumatoid arthritis (Batkhuu et al., 2002; Shen et al., 2002; Xia et al., 2004). Recently, several studies have reported that Andro exerts neuroprotective effects via inhibiting neuroinflammation and oxidative stress in neurodegenerative disorders. It has been reported that Andro could reduce the expression of NADPH oxidase 2 (NOX2), as well as inducible-nitric oxide synthase (iNOS), and it protect against cerebral ischemia via attenuating nuclear factor kappa B (NF-κB) and inhibiting the expression of hypoxia-inducible factor 1-α (HIF-1α) (Lu et al., 2006; Chern et al., 2011). Moreover, it has been reported that Andro reduced inflammation-mediated dopaminergic neurodegeneration by inhibiting microglial activation, indicating that Andro may be a new finding in clinical use in treating Parkinson’s disease (PD) (Wang et al., 2004, 2007). However, it remains unknown that whether Andro has impact on microglia-mediated inflammatory response in a model of TBI. Therefore, our main purpose of this work was to investigate neuroprotective effect of Andro after TBI and explore its underlying mechanism.

## Materials and Methods

### Animals and Treatment

Male adult Sprague–Dawley (SD) rats (300–350 g) were purchased from Animal Center of Chinese Academy of Sciences, Shanghai, China. The animals were housed in a temperature- and humidity-controlled room with a 12 h light/dark cycle and they were supplied with food and water *ad libitum*. All procedures of animal experiments were approved by the Institutional Animal Care Committee of the Soochow University and were according to the guidelines of the National Institutes of Health on the care and use of animals.

**FIGURE 1:**
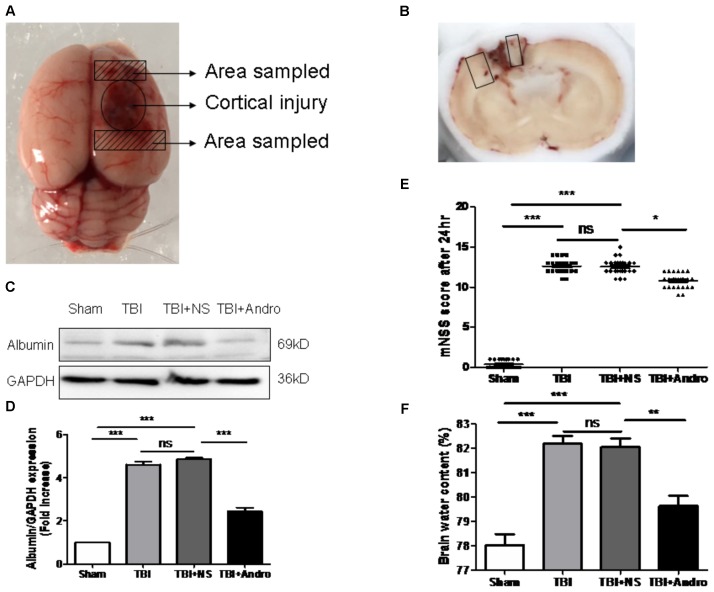
Andro treatment improved neurological function, reduced blood-brain barrier (BBB) disruption and cerebral edema after TBI. **(A)** A schematic image of the cortical contusion area induced by weight-dropping model of traumatic injury and the sampled area around the injured brain. **(B)** A schematic of a coronal section and the microphotographed areas used in immunofluorescence staining are marked with a box. **(C)** The expression level of albumin in peri-contusive cortex was examined by Western blot analysis at 24 h after TBI. **(D)** Relative expression levels of albumin were calculated based on densitometry analysis. The mean values of albumin in the sham group were normalized to 1.0. **(E)** Neurological function was measured by mNSS tests at 24 h after TBI. **(F)** Brain water content of each group was measured at 24 h after TBI. Data are presented as the mean ± SD. ^∗^*p* < 0.05, ^∗∗^*p* < 0.01 ^∗∗∗^*p* < 0.001, ns *p* > 0.05; *n* = 6 in each group.

A total 96 rats (118 rats were used, 96 rats were survived) were randomly divided into four groups: [(1) sham group (*n* = 24) (2) TBI group (*n* = 24) (3) TBI + saline (NS) group (*n* = 24) (4) TBI + Andro group (*n* = 24)]. [Mortality in the sham group is 0% (0 of 24), in TBI group is 25% (8 of 32), in TBI + NS group is 23% (7 of 31), and in TBI + Andro group is 23% (7 of 31)]. As described in previous study (Chern et al., 2011), Andro (Sigma-Aldrich) was first dissolved in ethanol, then it was diluted with 0.9% normal saline (NS). Rats were injected intraperitoneally with Andro (1 mg/kg) at 1 h after TBI model was conducted. The dose and administration method were chosen according to the previous study (Chan et al., 2010). Rats of TBI + NS group received an equal volume of saline, intraperitoneally. The brain areas around the injured cortex as shown in **Figures 1A,B** were collected for subsequent analysis.

### Experimental Model of Traumatic Brain Injury

Rat experimental model of TBI was established according to modified Feeney’s method (Feeney et al., 1981). Briefly, after shaving and sterilization, rats were fixed in a stereotactic frame, then a midline incision was made over the skull to expose the right parietal bone. A right 5 mm parietal craniotomy was drilled 2 mm caudal to the oronal suture and 3 mm from the midline. We modified the method of Feeney’s weight-drop model, an object weighing 40 g was dropped along a steel rod onto the dura from a height of 25 cm to cause severe brain injury (Feeney et al., 1981).

### Evaluation of Neurological Behavior Function

The modified neurological severity scores (mNSS) method was performed at 24 h to evaluate neurological function after TBI, a series of composite tests including motor, sensory, reflex and balance tests were measured and scaled from 0 to 18 score (Jin et al., 2014). The score of 0 means normal while 18 means severe brain injury.

### Brain Water Content

The brain edema of rats in each group was examined using a wet/dry method at 24 h post-injury. Briefly, bilateral brains were weighed separately as wet weight after quickly removed from the skull. The brains were dryed at 100 C in an oven for 72 h and weighed again as dry weight. The brain water content was calculated using the formula as follows: [(wet weight-dry weight)/wet weight] × 100% (Chen et al., 2017).

### Brain Tissue Preparation

For immunofluorescence staining, the rats in each group were sacrificed and perfused intracardially with NS and then fixed with 500 mL 4% paraformaldehyde at 24 h after TBI. The brains were quickly removed from skull and fixed with 4% paraformaldehyde. Then, the brains were dehydrated in 15%, 30% sucrose until infiltration was complete. For quantitative real-time PCR and Western Blot analysis, the rats were only infused with NS, then the brain tissues were quickly removed and the peri-contusive cortex were collected and stored at -80 C until use.

### Immunofluorescence Staining

The brains tissues were quickly removed from the rats, after embedded in paraffin and dehydration, brains tissues were sectioned at 15 μm. The staining for Iba1 (Wako Pure Chemical Industries, Osaka, Japan) was performed. Sections were first blocked with 5% bovine serum for 1 h and then incubated with primary antibodies at 4 C overnight. Sections were incubated with secondary antibodies (Life Technologies, United States) for 1 h after washing 3 times with PBST, After final washing, the sections were stained with DAPI (Southern Biotech, United States) for 30 min and observed in a fluorescent microscope (Leica, TCS SP8). Fluoro-Jade B (FJB) and terminal deoxynucleotidyl transferase (TdT) dUTP Nick-End Labeling (TUNEL) staining were performed to detect neuronal degeneration and apoptosis after TBI according to the manufacturer’s instructions. The relative fluorescence intensity was analyzed using Image J program.

### Quantitative Real-Time PCR

Quantitative real-time PCR analysis was performed to examine the gene expression levels of pro-inflammatory cytokines. Total RNA was extracted from peri-contusive cortex with the Trizol Reagents (Invitrogen Life Technologies, United States). cDNA was synthesized using a cDNA synthesis kit (Thermo Scientific) according to the manufacturer’s protocol. cDNA was amplified with SYBR Green (Thermo Fisher, United States) using specific primers of TNF-α, IL-6, IL-1β or GAPDH. The primer sequences were referred to our previous study (Tao et al., 2018). 2^-(ΔΔCT)^ method was choosed to calculate the quantification of relative gene expression and the values were normalized to GAPDH.

### Enzyme-Linked Immunosorbent Assay (ELISA)

The peri-contusive cortex were collected and homogenized. Then the supernatants were collected and centrifuged at 12,000 × *g* for 20 min. The expression of TNF-α, IL-1β and IL-6 were measured with ELISA kits (Boster Biosciences Co., Wuhan, China).

### Western Blot Analysis

The peri-contusive cortex were collected and lysed in RIPA buffer with protease inhibitors and the lysates were centrifuged at 12,000 × *g* for 15 min at 4 C. Protein concentration was determined using a BCA assay kit (Thermo Scientific). A total 80 μg protein was loaded on SDS–PAGE gel and transferred to polyvinylidene difluoride membranes (Millipore, MA, united States). Then the membranes were blocked with 5% fresh skimmed milk for 2 h, incubated with primary antibodies against albumin (Abcam Cambridge, United Kingdom), p38 MAPK (Abcam Cambridge, United Kingdom), ERK (Cell Signaling Technology, United States), NF-κB p65 (Cell Signaling Technology, United States) at 4 C overnight. After overnight incubation, the membranes were washed and incubated with secondary antibodies conjugated to horseradish peroxidase (Invitrogen Life Technologies, United States) for 1 h. Finally, protein bands were detected with enhanced chemiluminescence (ECL) reagents (Millipore, United States) in a Western Blotting Detection System. The relative protein quantity was analyzed by ImageJ software (National Institutes of Health, United States).

### Statistical Analysis

SPSS 19.0 software and GraphPad Prism were used in the experiment for data analysis. The data were presented as mean ± SD. Statistical significance was subjected to one-way ANOVA followed by Tukey’s test. *p* < 0.05 was considered statistically significant.

## Results

### Andro Attenuated Neurological Deficit, Cerebral Edema and BBB Breakdown in Rats Following TBI

Neurological function was confirmed using mNSS test at 24 h after injury in all groups of rats. Rat in TBI and TBI + NS group showed higher scores than sham group. As shown in **Figure 1E**, the scores of rats treated with Andro were decreased than that of TBI + NS group. Next, we detected the expression of albumin in each group to examine BBB breakdown after TBI. The results suggested that Andro treatment decreased the albumin expression induced by TBI (**Figures 1C,D**). Brain water content is an important indicator to in the prognosis after TBI. Our data showed that brain water content was markedly increased in the TBI and TBI + NS group compared with sham group. Andro treatment decreased brain water content 24 h after TBI (**Figure 1F**).

### Andro Inhibited Neuronal Apoptosis in Rats Following TBI

**FIGURE 2:**
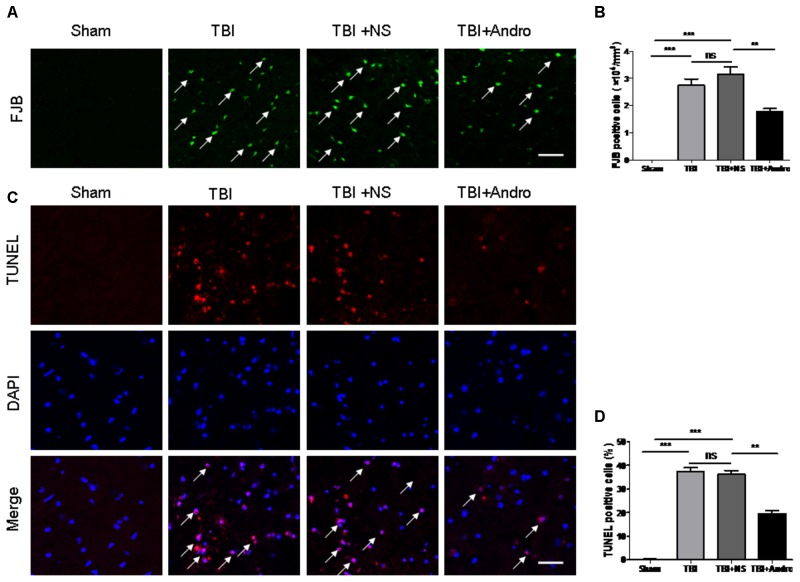
Andro treatment decreased the number of neuronal degeneration and apoptosis after TBI. **(A)** Degenerating neurons were detected with Fluoro-Jade B staining at 24 h after TBI. Arrows indicated the FJB-positive cells. **(B)** Percentage of FJB-positive cells around the injured brain tissues. **(C)** Apoptotic cells were labeled with TUNEL staining at 24 h after TBI. Arrows indicated the TUNEL-positive cells. **(D)** Percentage of TUNEL-positive cells around the injured brain tissues. Data are presented as the mean ± SD. ^∗^*p* < 0.05, ^∗∗^*p* < 0.01 ^∗∗∗^*p* < 0.001, ns *p* > 0.05; *n* = 6 in each group. Scale Bars = 50 μm.

Fluoro-Jade B staining, widely used as a marker of damaged neuronal cells, was performed to determine neuronal degeneration at 24 h after TBI. FJB staining is widely used as a maker of damaged neuronal cells (Ullah et al., 2011). The number of FJB-positive cells was significantly increased after injury, while Andro treatment decreased the number of FJB-positive cells (**Figures 2A,B**). Additionally, TUNEL staining was performed to detect neuronal apoptosis at 24 h after injury. As our results indicated that the number of TUNEL-positive cells was markedly increased after TBI and it was also decreased by Andro administration (**Figures 2C,D**). Both FJB and TUNEL staining revealed that Andro inhibited neuronal degeneration and apoptosis after TBI.

### Andro Inhibited Microglial Activation Following TBI

**FIGURE 3:**
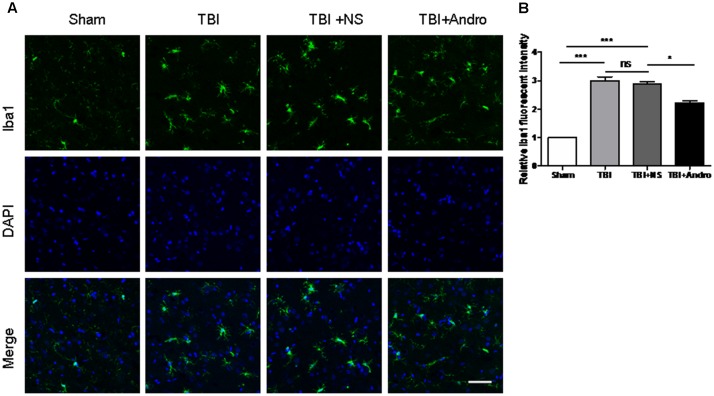
Andro treatment inhibited microglia activation after TBI. **(A)** Double staining for Iba1 (green) and 4, 6-diamino-2-phenylindole (DAPI, blue) in peri-contusive cortex at 24 h after TBI. Microglia cells were highly ramified, exhibited long branching processes and a small cellular body in the sham group. In response to brain injury, the branches of microglia become short, retracted and thick. In Andro treatment group, microglia showed a smaller cellular body. Scale Bars = 50 μm. **(B)** The relative fluorescent intensity of Iba1. Data are presented as the mean ± SD. ^∗^*p* < 0.05, ^∗∗^*p* < 0.01 ^∗∗∗^*p* < 0.001, ns *p* > 0.05; *n* = 6 in each group.

To investigate the effect of Andro on microglial activation, immunofluorescence staining of Iba1 was performed at 24 h after TBI. In the sham group, microglia cells were ramified with small cellular body, while microglia cells became thickened with larger bodies after injury. In Andro treatment group, cells showed a smaller cell body compared with TBI and TBI + NS group (**Figures 3A,B**).

### Andro Decreased the Production of Pro-Inflammatory Cytokines Following TBI

**FIGURE 4:**
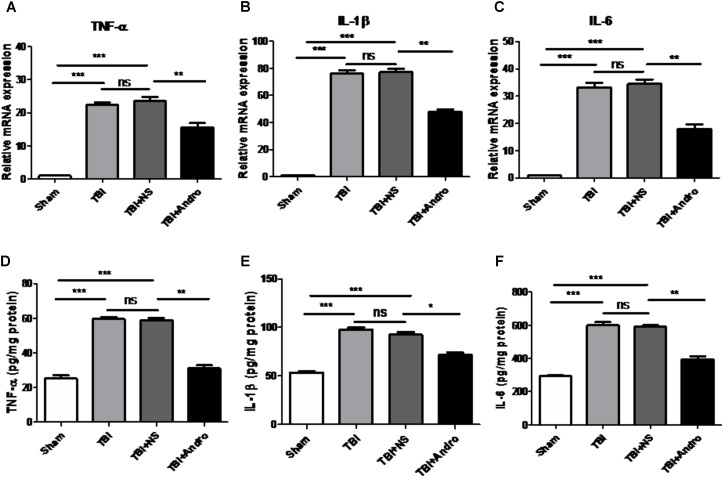
Andro treatment decreased the expression levels of pro-inflammatory cytokines after TBI. The mRNA levels of TNF-α **(A)**, IL-1β **(B)** and IL-6 **(C)** in peri-contusive cortex were determined by QPCR at 6 h after TBI. The protein levels of TNF-α **(D)**, IL-1β **(E)** and IL-6 **(F)** in peri-contusive cortex were measured by ELISA at 24 h after TBI. All data are presented as the mean ± SD. ^∗^*p* < 0.05, ^∗∗^*p* < 0.01 ^∗∗∗^*p* < 0.001, ns *p* > 0.05; *n* = 6 in each group.

The expression of TNF-α, IL-1β and IL-6 in the peri-contusive tissues were detected by qPCR and ELISA at 6 h and 24 h after TBI, separately. As shown in **Figure 4**, the expression of TNF-α, IL-1β and IL-6 were expressed at a low level in sham group. The expression of TNF-α, IL-1β and IL-6 was increased in TBI and TBI + NS group, while in the group of Andro treatment, the expression of pro-inflammatory cytokines were decreased.

### Andro Inhibited NF-κB Translocation and MAPK Activation Following TBI

**FIGURE 5:**
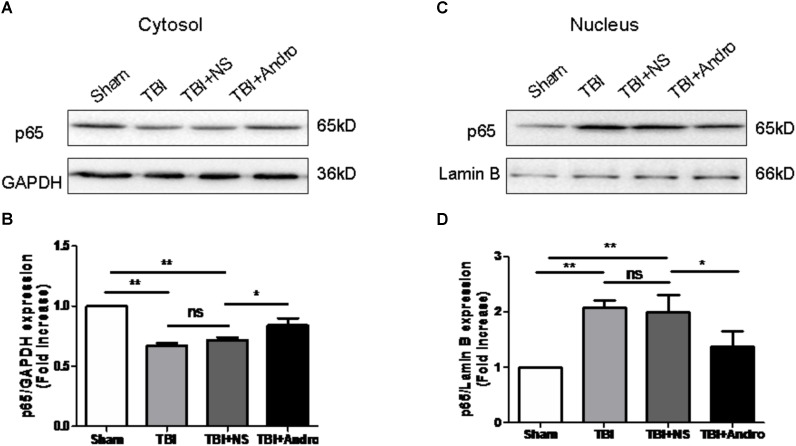
Andro treatment suppressed NF-κB translocation from the cytosol to the nucleus after TBI. Both cytoplasmic **(A)** and nuclear **(C)** protein were extracted in peri-contusive cortex at 24 h after TBI. The expression levels of NF-κB p65 were examined by Western blot analysis. Relative expression levels of NF-κB p65 in cytoplasmic **(B)** and nuclear **(D)** protein were calculated based on densitometry analysis. The mean values of NF-κB p65 in the sham group were normalized to 1.0. All data are presented as the mean ± SD. ^∗^*p* < 0.05, ^∗∗^*p* < 0.01 ^∗∗∗^*p* < 0.001, ns *p* > 0.05; *n* = 6 in each group.

**FIGURE 6:**
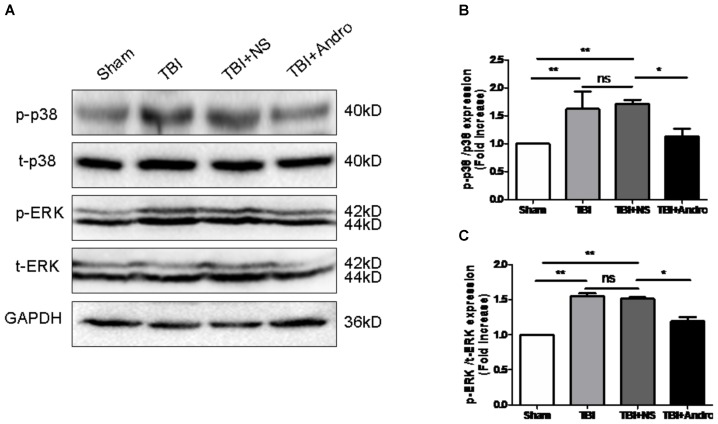
Andro treatment inhibited the phosphorylation of p38 MAPK and ERK after TBI. **(A)** The expression levels of p-p38 MAPK and p-ERK in peri-contusive cortex were examined by Western blot analysis at 24 h after TBI. Relative expression levels of p-p38 MAPK **(B)** and p-ERK **(C)** were calculated based on densitometry analysis. The mean values of *p*-p38 MAPK and *p*-ERK in the sham group were normalized to 1.0. All data are presented as the mean ± SD. ^∗^*p* < 0.05, ^∗∗^*p* < 0.01 ^∗∗∗^*p* < 0.001, ns *p* > 0.05; *n* = 6 in each group.

Both NF-κB and MAPK signaling pathway were involved in the modulation of pro-inflammatory cytokines (Tao et al., 2014). To investigate the underlying mechanism of the anti-inflammatory effect of Andro, we detected the expression of NF-κB and MAPK by Western blot analysis at 24 h after injury. As shown in **Figure 5**, Andro treatment inhibited p65 translocation from cytoplasm to nucleus after injury. Moreover, we determined the expression of p-ERK and p-p38 MAPK at 24 h after injury. The results of Western blot analysis suggested that Andro decreased the expression level of both p-ERK and p-p38 MAPK induced by TBI (**Figure 6**).

## Discussion

In this study, we demonstrated that Andro, a natural diterpenoid from Chinese traditional herb, conferred neuroprotective effect in rat model of TBI. Our data showed that Andro reduced brain edema, neuronal apoptosis and neurological deficit. In addition, Andro inhibited the expression of pro-inflammatory cytokines including TNF-α, IL-6 and IL-1β by QPCR and ELISA analysis. Moreover, Andro inhibited NF-κB p65 translocation from cytoplasm into the nucleus, the expression of p-ERK, p-p38 MAPK induced by TBI was also decreased after Andro treatment. To our knowledge, it is the first time we reported that Andro may attenuate acute neuroinflammation mediated by NF-κB and MAPK activation in acute phase of TBI.

It is well known that edema formation, increased oxidative stress, calcium influx, excitotoxicity, inflammation and cell death or apoptosis contribute to the process of secondary injury (Werner and Engelhard, 2007; Lutton et al., 2017). Brain edema exacerbation is a major and severe pathophysiological change induced by TBI (Lutton et al., 2017). In our study, we found that Andro reduced TBI-induced brain edema, moreover, Andro decreased the expression of albumin after injury, indicating that Andro rehabilitate the BBB integrity. Apoptosis plays a critical role in secondary brain injury following TBI. Neuronal apoptosis occurs as early as 4 h around the lesion site after injury and may last for weeks (Liu et al., 1997; He et al., 2018). Our results showed that Andro significantly reduced neuronal apoptosis and degeneration at 24 h after TBI.

Persistent neuroinflammation, characterized by glial cell activation, is associated with neurodegeneration and is an important mediator of progressive secondary injury (Chiu et al., 2016). Microglia cells exist in resting state in normal conditions, which is characterized by ramified morphology. When activated with stimuli, microglia cells became amoeboid and protected the damaged CNS (Morganti-Kossmann et al., 2007; Kadhim et al., 2008; Skaper et al., 2017, 2018). Activated microglia can produce and release a large number of pro-inflammatory cytokines and mediators, which can further exacerbate the inflammatory response and contribute to secondary brain injuries (Ding et al., 2014; Chen et al., 2017). Previous studies have been revealed that Andro inhibited microglia activation and decreased the production of pro-inflammatory chemicals in a rat model of stroke (Chan et al., 2010). However, the effects of Andro on TBI-induced microglial activation and the mechanism of Andro on neuroinflammation remain unknown. Our results indicated that in a rat model of TBI, microglia were activated at the acute phase of brain injury (24 h) and the expression of TNF-α, IL-1β and IL-6 were up regulated. After Andro administration, both microglia activation and the release of pro-inflammatory cytokines were inhibited, suggesting that Andro treatment could attenuate the microglial activation, reduce brain edema, ameliorate neuronal death and improve neurological function.

In order to further understand the mechanism by which Andro treatment inhibits TBI-induced inflammation, we detected the expression of relative factors involved in the NF-κB and MAPK signaling pathways. NF-κB activation is the first step in regulating inflammatory responses (Viatour et al., 2005; Zhang et al., 2018). Growing evidence has revealed that NF-κB signaling pathway also plays important role in TBI-induced inflammatory responses (Zhu et al., 2014; Chen et al., 2017; Zhao et al., 2017). Previous studies have demonstrated that Andro could decrease the production of cytokines including TNF-α and IL-1β, and pro-inflammatory factors such as PGE2 through inhibiting NF-κB activation to protect against cerebral ischemia (Chan et al., 2010; Chern et al., 2011). Moreover, it has been reported that Andro reduced inflammation-mediated dopaminergic neurodegeneration by inhibiting microglial activation, indicating that Andro may be a new finding in clinical use in treating Parkinson’s disease (PD). However, the mechanism of its anti-inflammatory effects in TBI model remains unknown. In our present study, Andro treatment suppressed not only production of pro-inflammatory cytokines but also NF-κB translocation from the cytosol to the nucleus. Mitogen-activated protein kinases (MAPKs), a family of serine/threonine protein kinases, are composed of three members: ERK, JNK and p38 MAPK. The MAPK signaling pathway is one of the major pathways involved in regulating inflammatory responses (Luo et al., 2013). Here, we found that Andro inhibited NF-κB p65 translocation and suppressed the expression of p-p38 MAPK and p-ERK. These results revealed that Andro could inhibit inflammatory responses after TBI via NF-κB and MAPK pathway.

It is our first time to demonstrate that Andro had neuroprotective effects in TBI. However, there exist some limitations about this study. We demonstrated the neuroprotective effects of Andro in early time point (24 h) after TBI, further investigations are needed to illuminate the effect of Andro in long-term recovery processes. Taken together, it is likely that Andro exhibited neuroprotective effects through inhibiting inflammation by blocking the NF-κB and MAPK activation in TBI.

## Author Contributions

HL and JC conceived and designed the study. LT and LZ acquired the data and drafted the manuscript. RG and FJ contributed to data analysis and interpretation.

## Conflict of Interest Statement

The authors declare that the research was conducted in the absence of any commercial or financial relationships that could be construed as a potential conflict of interest.
